# Exfoliated Kidney Cells from Urine for Early Diagnosis and Prognostication of CKD: The Way of the Future?

**DOI:** 10.3390/ijms23147610

**Published:** 2022-07-09

**Authors:** Henry H. L. Wu, Ewa M. Goldys, Carol A. Pollock, Sonia Saad

**Affiliations:** 1 Renal Research Laboratory, Kolling Institute of Medical Research, The University of Sydney, Sydney, NSW 2065, Australia; howu5814@uni.sydney.edu.au (H.H.L.W.); carol.pollock@sydney.edu.au (C.A.P.); 2 School of Biomedical Engineering, The University of New South Wales, Sydney, NSW 2052, Australia; e.goldys@unsw.edu.au

**Keywords:** exfoliated kidney cells, chronic kidney disease, non-invasive, early diagnosis, prognostication

## Abstract

Chronic kidney disease (CKD) is a global health issue, affecting more than 10% of the worldwide population. The current approach for formal diagnosis and prognostication of CKD typically relies on non-invasive serum and urine biomarkers such as serum creatinine and albuminuria. However, histological evidence of tubulointerstitial fibrosis is the ‘gold standard’ marker of the likelihood of disease progression. The development of novel biomedical technologies to evaluate exfoliated kidney cells from urine for non-invasive diagnosis and prognostication of CKD presents opportunities to avoid kidney biopsy for the purpose of prognostication. Efforts to apply these technologies more widely in clinical practice are encouraged, given their potential as a cost-effective approach, and no risk of post-biopsy complications such as bleeding, pain and hospitalization. The identification of biomarkers in exfoliated kidney cells from urine via western blotting, enzyme-linked immunosorbent assay (ELISA), immunofluorescence techniques, measurement of cell and protein-specific messenger ribonucleic acid (mRNA)/micro-RNA and other techniques have been reported. Recent innovations such as multispectral autofluorescence imaging and single-cell RNA sequencing (scRNA-seq) have brought additional dimensions to the clinical application of exfoliated kidney cells from urine. In this review, we discuss the current evidence regarding the utility of exfoliated proximal tubule cells (PTC), podocytes, mesangial cells, extracellular vesicles and stem/progenitor cells as surrogate markers for the early diagnosis and prognostication of CKD. Future directions for development within this research area are also identified.

## 1. Introduction

Chronic kidney disease (CKD) is a progressive disease that is defined by structural and functional changes to the kidney [1]. CKD is considered to be a global issue, one with a substantial public health burden which is exponentially growing [2]. With more than 10% of the adult population currently affected by CKD, it is projected to become the fifth leading cause of mortality worldwide by 2040 [3]. There are multiple causes of CKD, some of which are more common and clearly defined (e.g., diabetes mellitus, hypertension, glomerulonephritis and polycystic kidney disease), whilst others are not fully understood (e.g., Mesoamerican nephropathy) [4,5,6,7]. CKD progresses differently in each individual, depending on the primary cause of CKD, as well as other co-morbidities [8]. CKD is typically identified by a reduction in kidney function, an estimated glomerular filtration rate (eGFR) of less than 60 mL/min/1.73 m^2^, and supported by markers of kidney tissue damage (albuminuria and hematuria), as well as other laboratory-based and imaging investigations that are present for at least 3 months [9]. The identification of early CKD, particularly in younger patients, remains challenging. Asymptomatic individuals living with CKD can lose up to 90% of their kidney function, at which point CKD is irreversible, given the advanced pathological damage [10]. Early diagnosis of CKD is clinically important, given that therapies are now available to stabilize kidney function from an early stage of the disease [11,12].

Histopathological examination of the kidney is the gold standard for the diagnosis and prognostication of CKD [13]. However, the risk of adverse events for patients after kidney biopsy has been well-documented. Post-biopsy risks include bleeding, excess pain and occasionally nephrectomy. For most individuals, bleeding usually resolves spontaneously following kidney biopsy, although for a small percentage of individuals, blood transfusion may be required [14,15]. Recently, the stress for the physician of routinely performing kidney biopsies has been addressed, with the increasing workload and time pressures of the modern-day clinical environment contributing to this issue [16]. There is a suggestion that the quality of training in kidney biopsy has reduced in recent years [17]. With the continuous development of non-invasive diagnostic and prognostication tools in CKD, it is questioned whether other diagnostic methods can complement or replace kidney biopsy in the near future.

A reliable non-invasive method to detect early kidney fibrosis in CKD, and to predict the trajectory of CKD progression remains desirable. As a disease marker, microalbuminuria has long been considered to be the potential solution, but it has since demonstrated to be non-specific in the detection of early CKD [18]. Following an initial report of cell culture success from urine in newborn children in 1972 by Sutherland and Bain, exfoliated kidney cells from urine have emerged as a potentially useful source to non-invasively diagnose early CKD and prognosticate CKD progression [19]. Exfoliation is an active biochemical process that is linked to the homeostasis of epithelial cells in mammalian organs such as the gut and placenta, as well as the kidney [20]. It is considered to play a significant role in preserving the epithelial layer’s architectural integrity, with this being a natural process where external cells are removed from the epithelial luminal surface to ensure the epithelium remains structurally intact and primed for further growth [20]. Exfoliated cells into the urine are most likely a single cell, or a group of cells from the epithelial layer which can be detached from tissue [20]. Given the passage of urine prior to excretion, some of these exfoliated cells would be sourced from the nephron and can generate useful information regarding the kidney’s histopathological status. Small amounts of senescent epithelial cells are usually observed in exfoliated cells from the urine of healthy individuals [21]. On the other hand, an increased amount of exfoliated cells from the urine is expected in individuals with active kidney disease [21].

Difficulties in the maintenance of exfoliated kidney cells from urine are acknowledged, as mature exfoliated cells have a short lifespan in culture [22]. Immortalization techniques play an instrumental role in culture to extend cell survival. Together with the advancement of biomedical technology, this led to improved quality in the processes of cell isolation and characterization for exfoliated kidney cells from urine [21,23,24]. Numerous techniques of cell isolation and characterization have been innovated in recent years, allowing exfoliated cells from the urine to be used as surrogate markers for biopsied tissue in predicting changes relating to gene expression, deoxyribonucleic acid (DNA) methylation, DNA damage and protein expression in the kidney [22,25,26,27,28]. The application of exfoliated kidney cells from urine into clinical practice has increased as a result.

Our review evaluates the various applications of exfoliated proximal tubule cells (PTC), podocytes and extracellular vesicles (EV) from urine in CKD. The potential application of exfoliated stem/progenitor kidney cells from urine is also discussed.

## 2. Exfoliated Podocytes

Urinary exfoliated podocytes and podocyte-specific markers have demonstrated value for the early diagnosis of CKD and prognosticating CKD progression (Table 1). Diabetic kidney disease (DKD) is the most common cause of CKD worldwide. In a post-hoc exploratory analysis comparing archived urine samples from normoalbuminuric patients with uncomplicated type 1 diabetes and healthy controls, urinary podocyte microparticle levels were found to be higher in the cohort with type 1 diabetes [29]. Interestingly, the elevation of urinary podocyte microparticle levels was well in advance of changes to other more well-established biomarkers of CKD such as albuminuria and nephrin, suggesting its potential utility as an early biomarker of glomerular injury in uncomplicated type 1 diabetes [29]. There is evidence demonstrating significant differences in urinary podocyte mRNA levels of nephrin, podocin, synaptopodin, Wilms Tumor-1 (WT-1) and α-actinin-4 between DKD and non-DKD patients [30]. These markers were found to precede the clinical appearance of microalbuminuria in patients with type 2 diabetes [31]. Urinary synaptopodocin mRNA levels were used to measure therapeutic response to angiotensin-converting enzyme inhibitor and angiotensin-receptor blocker treatment in DKD [32]. Urinary podocyte-derived indices such as podocin mRNA-to-creatinine ratio are a strong marker of podocyte detachment from GBM and are shown to project the rate of kidney functional decline in DKD [30]. For minimal change disease (MCN) and focal segmental glomerulosclerosis (FSGS), urinary nephrin and podocin mRNA levels were lower in patients with MCN and FSGS compared to healthy controls, and urinary nephrin and podocin mRNA levels correlated with the degree of proteinuria within this context [33]. Urinary synaptopodin mRNA levels were found to correlate with kidney function decline in FSGS [33]. In membranous nephropathy (MN), the number of urinary podocyte-derived microparticles displayed an inverse relationship with clinical parameters of MN, decreasing with improving clinical parameters following immunosuppression treatment [34]. The urinary podocyte mRNA levels of nephrin, podocin, and synaptopodin were all elevated in MN, with these levels clearly differentiating MN from other causes of nephrotic syndrome [35].

The role of exfoliated podocytes from urine as markers to prognosticate the progression of mesangial diseases such as IgA nephropathy has been explored. Previous studies noted that urinary podocyte counts correlated with serum creatinine and proteinuria in IgA nephropathy [36]. Those with segmental sclerosis, which was confirmed histologically, had greater numbers of urinary podocytes compared to those without segmental sclerosis [37]. Evidence is incomplete regarding the use of podocyte-specific mRNA and miRNA levels in IgA nephropathy as biomarkers for early diagnosis and prognostication, and this requires further study. For other mesangial glomerulopathies outside of IgA nephropathy, urinary podocyte counts are increased in hereditary and acquired diffuse mesangial sclerosis [37]. The application of non-invasive risk assessment techniques is not as well studied for these conditions, likely explained by a lack of clarity in pathological classification.

Utilizing urinary podocytes as markers of disease activity in lupus nephritis has been evaluated. Studies have noted that the majority of urinary podocytes in patients with lupus nephritis are viable but dedifferentiated, with a greater proportion of apoptotic urinary podocytes being much lower in patients without kidney disease [38]. Urinary podocyte-specific mRNA markers such as podocalyxin, synaptopodin, podocin, nephrin, as well as WT-1 levels, are significantly elevated in patients with active lupus nephritis compared to those without systemic lupus or active lupus nephritis [39]. Within this context, urinary nephrin mRNA levels are shown to correlate with the degree of proteinuria and systemic lupus disease activity, but not with the histological progression of lupus nephritis [42]. Meanwhile, urinary podocin mRNA levels are demonstrated to be an independent predictor of kidney function decline in lupus nephritis [38,39,42].

There is minimal data regarding the use of exfoliated podocytes from urine for anti-neutrophil cytoplasmic antibody (ANCA)-associated vasculitis. It was previously found that the rate of podocyte detachment to urine predicted kidney function loss in ANCA-associated vasculitis [43]. Reports have also suggested that urinary podocin-to-nephrin mRNA ratio, a surrogate marker of intra-glomerular podocyte stress, correlated with the extent of crescent formation [40]. Paradoxically, patients with higher urinary podocyte-specific mRNA levels achieved better outcomes, as this indicated a stronger glomerular podocyte reserve for the reversibility of vasculitic disease [40].

Ultimately, there are urinary podocyte-specific biomarkers which have shown universal prognostic value for all forms of CKD. Urine synaptopodin levels are described as a generic marker of podocyte damage. Synaptopodin protein expression, determined by Western blot, has demonstrated significant correlations with kidney function in CKD, regardless of the degree of albuminuria [44]. Urinary podocyte-specific mRNA targets such as urinary brain-derived neurotrophic factor mRNA level had the best correlation with urinary kidney injury molecule-1 (KIM-1), which is recognized as a generic marker to prognosticate CKD progression [41].

The recent development of single-cell RNA sequencing (scRNA-seq) is a revolutionary technique in providing an unbiased genome-wide characterization of individual exfoliated cells from urine at scale [45]. Both animal and human studies have demonstrated that scRNA-seq is able to generate an initial map of gene expression for most kidney cells, thereby allowing us to advance from a morphology-based cell characterization through cell shape, color and location to the more objective method of cellular definition through transcriptomics [46,47]. In CKD, scRNA-seq may be able to define cell type-specific changes, cell fractions and cell-to-cell interactions [26,48,49]. This information can be useful for the diagnosis and risk stratification of CKD. In a combined analysis between urinary exfoliated podocytes, bladder single cells and human kidney tissue nucleus (extracted from DKD and control patients) scRNA-seq datasets, a strong correlative relationship was found between exfoliated podocyte and kidney tissue nucleus scRNA-seq datasets, where together they formed a strong cluster [26]. Urinary scRNA-seq has particularly shown a strong expression of monogenic nephrotic syndrome genes in podocytes [26]. Nevertheless, these results were obtained in a pilot study with few patients and controls. Abedini and colleagues ensured urine was collected at different time points and through different methods to confirm the reproducibility and feasibility of this approach [26]. Other confounding factors include the differences of scRNA-seq in capturing efficiency between the male and female urine samples, and perhaps a subgroup analyses between them is required to reduce sex-associated bias in the interpretation of results. Furthermore, there was significantly higher ambient RNA contamination in 24-h urine collections [26]. Meticulous arrangement to optimize the environment of urinary cell storage may mitigate the risks of cellular degradation during extended storage. As the authors noted, large prospective cohort studies are needed to validate the diagnostic and prognostic utility of urinary scRNA-seq in CKD.

## 3. Exfoliated Proximal Tubule Cells

The utility of exfoliated PTCs from urine has proven to be clinically valuable in prognosticating the degree of disease for various tubular diseases that are associated with CKD, including conditions with a genetic predisposition [22,50,51]. It is suggested that exfoliated PTCs from urine may represent the degree of pathology in the kidneys [22,52]. Previous studies noted the number of exfoliated PTCs correlating with kidney function decline in DKD, though this may not correspond to the degree of kidney injury in all cases [52,53]. Exfoliated PTCs from urine have also been used in in vitro studies evaluating tubular cell toxicology in CKD, in order to measure drug influx and efflux in tubular cells [54]. Nevertheless, the use of exfoliated PTCs from urine for early diagnosis and risk stratification in CKD have not been as widely assessed compared to podocytes. This is mainly due to a limited number of exfoliated PTCs in the urine, as well as the short life span and the weak proliferative ability of exfoliated PTCs in cell culture. Our CELLection Pan anti-mouse dynabeads methodology for PTC isolation presents an alternative cell isolation technique to avoid being reliant on culturing urinary exfoliated PTCs [52]. This technique is based on a specific antibody and magnetic beads selection methodology using CD13 and sodium-glucose linked transporter-2 antibodies, and we have shown that isolated cells express angiotensinogen as well [52]. Aquaporin-1 is also a highly specific marker which is positive on PTCs, along with other markers such as N-Cadherin and CD10 [55,56].

Differential multispectral autofluorescence imaging is an innovative technique that may have the potential to non-invasively diagnose CKD from an early stage and to prognosticate CKD progression. Cell autofluorescence originates from native fluorophores (collagen, elastin, tryptophan, reduced levels of nicotinamide adenine dinucleotide and flavins) that play important roles in cell and tissue metabolism [57,58]. The use of multispectral microscopy can collect native emission data across a broad range of excitation wavelengths. Cell autofluorescence features which define each cell’s spectral profile, including parameters from average channel intensity, channel intensity ratio, pixel standard deviations to skewness could be obtained via multispectral microscopy [59]. This may provide a fingerprint that can be used to distinguish the cellular and metabolic characteristics of each cell, from their cell cycle stage, inflammatory state, extent of oxidative stress, presence of neoplasia and degree of senescence [60,61,62,63,64]. We have shown that a multispectral assessment of cell autofluorescence is highly sensitive towards the identification of metabolic changes and oxidative stress [60,62]. We have additionally demonstrated that the multispectral autofluorescence imaging of exfoliated PTCs from urine may have high diagnostic value in reflecting kidney pathology due to CKD [52]. Using ten features, the technique was able to differentiate cells between individuals with normal and impaired kidney function (normal kidney function was defined by eGFR ≥60 mL/min/1.73 m^2^; impaired kidney function was defined by eGFR <60 mL/min/1.73 m^2^) with a receiver operating characteristic area under the curve (AUC) of 0.99 [52]. Furthermore, exfoliated PTCs from urine between patients with and without tubulointerstitial fibrosis on kidney biopsy could be discriminated using this technique, where significant differences in multispectral autofluorescence signals were observed [52]. Further investigation needs to be undertaken to determine whether changes in exfoliated PTC autofluorescence can reflect CKD stages and is reliable enough to diagnose early CKD pathology when serum biomarkers for kidney disease appear within a normal range, or to prognosticate future CKD progression.

Another method involving the use of exfoliated PTCs from urine as a risk stratification tool in CKD is through proximal tubule-specific DNA methylation patterns that are identified from compartment-specific methylome analysis [25]. These DNA methylation patterns from exfoliated PTCs in the urine correlated with kidney function decline in patients with DKD [25]. The original study identified genomic loci in SMTNL2 and G6PC to be selectively unmethylated in human PTCs [25]. SMTNL2 and G6PC methylation levels appeared to correlate to the extent of injury in exfoliated PTCs from urine, in which SMTNL2 methylation levels significantly correlated with the annual decline in eGFR [25]. The addition of urinary SMTNL2 methylation to a multivariate model (which adjusted for eGFR and albumin:creatinine ratio amongst other parameters) significantly improved the discrimination of patients with DKD and faster rates of eGFR decline [25]. Early results advocate for the clinical potential of a screening urine test to detect CKD from an early stage, based on a principle of quantifying exfoliated PTCs from their specific DNA methylation patterns. The use of epigenetic urinalysis to determine kidney cell type-specific DNA methylation in urine allows for the site-specific monitoring of kidney cell turnover activity, and enhances this method to achieve early detection of CKD [25].

The application of scRNA-seq in exfoliated PTCs from urine has shown potential utility as a non-invasive technique for early diagnosis and risk stratification in CKD [26]. The comparison of a human urine single-cell dataset with kidney single nucleus and bladder scRNA-seq datasets, and the combined analysis of urine, bladder single-cell and human kidney nucleus datasets indicated that there were multiple PTC subclusters, with numerous subclusters demonstrating severe de-differentiation [26]. Exfoliated PTC cells from urine clustered better with biopsied PTC cells that were profiled in patients with DKD compared to those with healthy kidneys [26]. As scRNA-seqs have shown a better ability to distinguish between the specific cell subtypes that are exfoliated from the proximal tubule compared to other techniques, this information may be important to follow when monitoring CKD progression over the long-term. It should be considered however, that the costs of performing urinary scRNA-seq over the long-term may be a hindering factor. Furthermore, urinary scRNA-seq illustrated that the expression of genes nominated to mediate the effect of the polygenic eGFR genome-wide association studies also had strong enrichment for the expression of exfoliated PTCs from urine [26]. This supports the use of scRNA-seq techniques to guide the collation of diagnostic and prognostic information in CKD.

There are promising urinary biomarkers (e.g., KIM-1, monocyte chemoattractant protein-1 and neutrophil gelatinase-associated lipocalin) which reflect proximal tubule injury, inflammation and fibrosis in CKD, although it remains uncertain whether these biomarkers could be reliably expressed in urinary exfoliated PTCs [65,66]. Further work needs to be undertaken to determine if these biomarkers could be extracted from urinary exfoliated PTCs, and the extent to which they are useful for prognosticating the diverse etiologies of CKD.

## 4. Extracellular Vesicles from Exfoliated Kidney Cells

Markers that are derived from the EVs of urinary exfoliated kidney cells have demonstrated diagnostic and prognostic value for various etiologies of CKD (Table 2). GSK-3β is an exosomal enzyme found in exfoliated kidney cells (mostly podocytes) from urine, which has shown greater prognostic accuracy to determine DKD progression when compared with albuminuria [67]. Previous studies demonstrated that exosomal WT-1 from urine correlated with the severity of proteinuria, extent of glomerular damage and rate of kidney function decline in diabetic patients [68,69]. In DKD, urinary microvesicle-dipeptidyl peptidase-IV level correlates with urinary albumin-to-creatinine ratio and regucalcin levels are reduced [70]. High levels of EV podocalyxin or a high podocin-to-nephrin ratio is suggestive of glomerular injury in DKD [71]. Further work is currently being undertaken to evaluate which are the best EV biomarkers to diagnose early DKD and prognosticate accurately from an early stage, and how these markers could be affected by glycosuria or the presence of other protein complexes [72]. When significant hypertension presents with CKD, significantly elevated numbers of urinary EV markers such as nephrin, podocalyxin, urate-transporter-1+/p16 suggestive of proximal tubule senescence, and plasmalemmal vesicle-associated protein reflecting microvascular injury in the kidneys are observed [43,73,74]. Other key markers include exosomes miR-21 and miR-146a [75,76]. Previous studies have shown increased urinary exosomal miR-21 levels in animal models and patients with CKD, and correlated with the severity of podocyte injury [75]. Further studies are needed to determine the cellular origins of miR-21. Exosome miR-146a has inverse associations with albuminuria and early kidney cell damage, making it valuable in the process of identifying early CKD, prior to albuminuria, for early intervention [76]. In conditions such as IgA nephropathy, elevated levels of C-C motif, chemokine ligand-2 mRNA40, α-1-antitrypsin, and ceruloplasmin, as well as reduced aminopeptidase and vasorin precursor, are typically observed [77]. For genetic conditions such as polycystic kidney disease, polycystin/transmembrane protein-2 ratio in urinary EVs is noted to be a useful diagnostic and prognostic marker [78]. Decreased levels or the total absence of CD133 has been observed in kidney failure, and where significant glomerular injury is indicated [79]. For glomerulonephritides such as lupus nephritis, urinary EVs such as annexin-V and podocalyxin display a correlating relationship with disease activity, with other miRNAs also suggested as biomarkers [80]. Ongoing studies are required to clarify their selectivity for the pathogenesis of lupus nephritis. Decreased levels of vacuolar adenosinetriphosphatase-B1 have been shown to detect distal tubular acidosis [81]. miR-26a and WT1 mRNA have recently been shown to associate with podocytopathy [82,83]. The role of lipid metabolites in urinary EVs as biomarkers for CKD is less clear compared to that of protein and nucleic acid, though their use for renal cell carcinoma and prostate cancer has been studied in greater detail previously [84,85,86]. Additional research is needed to determine their use for diagnosis and risk stratification in CKD.

## 5. Exfoliated Stem/Progenitor Kidney Cells

An advancement in our understanding of podocyte development has increased interest in the potential clinical applications of stem/progenitor cells in adult kidneys, though few studies have explicitly explored the marker utility of stem/progenitor cells exfoliated from human urine for the early diagnosis and risk stratification of CKD. Urinary stem/progenitor kidney cells have been quantified in genetic kidney disease studies, appearing with increased numbers over time in conditions such as cystinosis, mainly in the presence of proteinuria [87,88,89]. The increased number of stem/progenitors cells in urine might indicate an attempt of tissue regeneration, which may turn out to be a maladaptive process resulting in further CKD progression [88,89]. Therefore, the evaluation of stem/progenitor kidney cells in urine could be useful for the early diagnosis of CKD in this respect. In another study, Wang and colleagues isolated and sequenced 2200 cells from urine-derived cell suspensions through a scRNA-seq analysis of voided urine samples from 12 healthy adults, in which 1100 cells were analyzed using stringent quality controls [90]. The authors identified a SOX9 cell population which was speculated to have progenitor potential [90]. Further prospective study with larger cohorts combined with healthy individuals and patients with CKD are needed, in order to explore optimal methods of identifying exfoliated stem/progenitor cells in urine, and to determine the diagnostic and prognostic value of the potential markers that are derived from these exfoliated cells.

## 6. Summary and Future Directions

**Figure 1:**
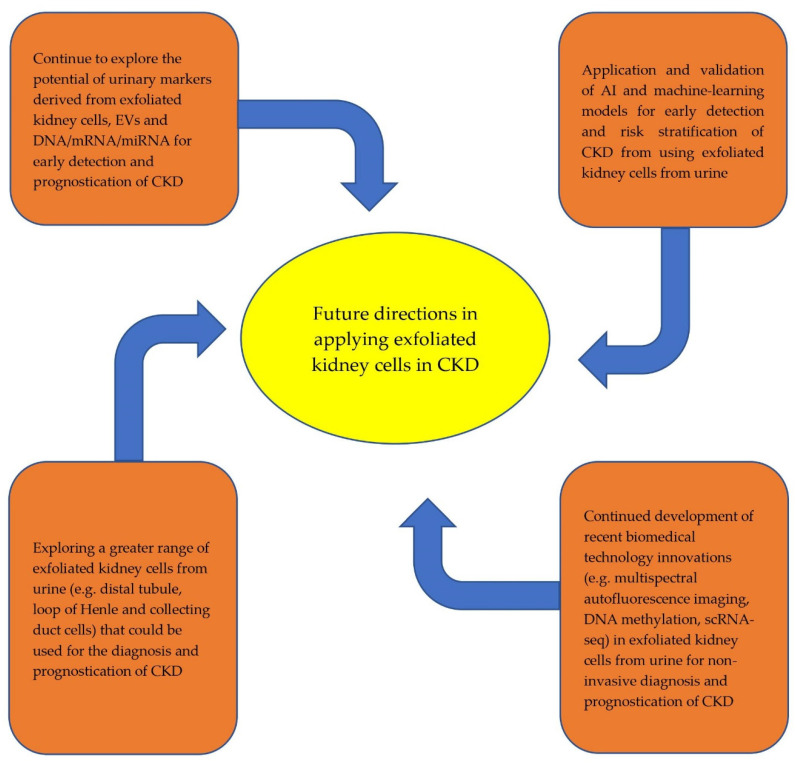
Future directions in the application of exfoliated kidney cells from urine for early diagnosis and prognostication of CKD.

Recent developments in novel technologies to utilize the clinical application of exfoliated kidney cells from urine have been encouraging. There is increased evidence to support the use of these non-invasive techniques for the early diagnosis and prognostication of CKD. Future directions in which to expand research on this topic are exciting (Figure 1).

Whilst there are growing data to support the clinical use of exfoliated PTCs, podocytes, extracellular vesicles and stem/progenitor cells for the early diagnosis and prognostication of CKD, such data have been mostly absent for other structures in the nephron, such as mesangial cells, distal tubule, loop of Henle and collecting duct cells. ScRNA-seq techniques suggest that these cells can be exfoliated from urine, and EV biomarkers have been found in these cells with kidney disease [26,81,91,92,93]. Further study is needed to ascertain whether these exfoliated cells are of clinical value for the early diagnosis and prognostication of CKD. Opportunities to expand on novel technological approaches in this context beyond recent developments of differential multispectral autofluorescence imaging, DNA methylation and scRNA-seq techniques are vast. Artificial intelligence (AI) and machine-learning innovations that are applied to exfoliated kidney cells from urine have seen tremendous progression, particularly their use in acute kidney injury and post-transplantation scenarios to augment decision-making regarding clinical management and to prognosticate outcomes [94,95,96,97]. Within the CKD context, AI and machine-learning models have been created for use as early identification and clinical decision aids in DKD, using information from electronic health records and biomarkers, though these models are not yet externally validated [98]. There are concerns regarding the under-prediction of all quantities of risk during the internal validation of these models [99]. We anticipate a greater focus on improving the practicality of these technologies going forward.

## Figures and Tables

**Table 1:** Clinical utility of urinary exfoliated podocytes and podocyte-specific markers for early diagnosis and prognostication of CKD.

Etiology of CKD	Clinical Utility of Urinary Exfoliated Podocytes and Podocyte-Specific Markers
DKD	Exfoliated podocyte microparticles in early DKD may prognosticate kidney function decline and disease progression [29] ELISA of nephrin, podocalyxin and PCR-based testing of nephrin, podocin, synaptopodin mRNA, WT-1 and α-actinin 4 in DKD can identify early disease, assess degree of podocyte loss and monitor treatment response [30,31,32] Podocin mRNA-to-creatinine ratio is a strong podocyte-derived index of podocyte detachment from GBM, and is shown to project the rate of kidney functional decline in DKD [30]
MCN and FSGS	PCR-based testing of nephrin and podocin mRNA in MCN and FSGS to differentiate between MCN and FSGS, nephrin and podocin mRNA levels correlated with the degree of proteinuria in MCN and FSGS [33] PCR-based testing of urinary synaptopodin mRNA levels found correlations with kidney function decline in FSGS [33]
MN	Exfoliated podocyte microparticles can monitor treatment response in MN [34] Urinary podocyte-specific nephrin, podocin, and synaptopodin all usually markedly elevated in MN following PCR-based testing. These markedly elevated levels allow for differentiation of MN from other causes of nephrotic syndrome [35]
IgA nephropathy	Indirect IF of exfoliated podocyte count in IgA nephropathy can be utilized to prognosticate histological severity [36,37]
Lupus nephritis	A greater proportion of apoptotic urinary exfoliated podocytes in patients with lupus nephritis compared to those without is typically found following flow cytometry [38] Western blotting of nephrin, podocin, WT-1, podocalyxin, synaptopodocin and PCR-based testing of nephrin, podocin, synaptopodocin mRNA in lupus nephritis to prognosticate disease severity and kidney function decline [39]
ANCA-associated vasculitis	PCR-based testing of podocin mRNA in ANCA-associated vasculitis to assess histological severity and predict disease progression [40] Urinary podocin-to-nephrin mRNA ratio, a surrogate marker of intra-glomerular podocyte stress, correlated with the extent of crescent formation in ANCA-associated vasculitis [40]
General CKD	Western blotting of urinary podocyte-specific synaptopodin protein expression demonstrated significant correlations with kidney function in all forms of CKD, regardless of the degree of albuminuria [41] Application of scRNA-seq techniques for exfoliated podocytes for early identification and prognostication of CKD require further development [26]

ANCA: anti-neutrophil cytoplasmic antibody; CKD: chronic kidney disease; DKD: diabetic kidney disease; ELISA: enzyme-linked immunosorbent assay; FSGS: focal segmental glomerulosclerosis; GBM: glomerular basement membrane; IF: immunofluorescence; IgA: immunoglobulin A; MCN: minimal change disease; mRNA: messenger ribonucleic acid; MN: membranous nephropathy; PCR: polymerase chain reaction; scRNA-seq: single cell ribonucleic acid sequencing; WT-1: Wilms Tumor-1.

**Table 2:** Clinical utility of extracellular vesicles from exfoliated kidney cells for early diagnosis and prognostication of CKD.

Etiology of CKD	Clinical Utility of Extracellular Vesicles from Exfoliated Kidney Cells
DKD	The exosomal enzyme GSK-3β found in exfoliated kidney cells (mostly podocytes) from urine shows greater prognostic accuracy to determine DKD progression when compared with albuminuria [67] Exosomal WT-1 from urine correlates with the severity of proteinuria, extent of glomerular damage and rate of kidney function decline in diabetic patients [68,69] Urinary microvesicle-dipeptidyl peptidase-IV level correlates with urinary albumin-to-creatinine ratio and regucalcin levels are reduced in DKD [70] High levels of EV podocalyxin or a high podocin-to-nephrin ratio is suggestive of glomerular injury in DKD [71]
Tubule disease	Urinary EV markers such as nephrin, podocalyxin, urate-transporter-1+/p16 suggestive of proximal tubule senescence, and plasmalemmal vesicle-associated protein reflecting microvascular injury in the kidneys are observed when there is hypertensive disease with CKD [43,73,74] Decreased levels of vacuolar adenosinetriphosphatase-B1 have been shown to detect distal tubular acidosis [81]
IgA nephropathy	Elevated levels of C-C motif, chemokine ligand-2 mRNA40, α-1-antitrypsin, and ceruloplasmin, as well as reduced aminopeptidase and vasorin precursor are typically observed in IgA nephropathy [77]
Polycystic kidney disease	Polycystin/transmembrane protein-2 ratio in urinary EVs is noted to be a useful diagnostic and prognostic marker for polycystic kidney disease [78]
Lupus nephritis	Urinary EVs such as annexin-V and podocalyxin display a correlating relationship with disease activity in lupus nephritis [80]
General podocytopathies/CKD	Urinary exosomal miR-21 levels in animal models and patients with CKD correlated with the severity of podocyte injury [75,76] Decreased levels or a total absence of CD133 has been observed in kidney failure, and where significant glomerular injury is indicated [79] PCR-based testing of miR-26a and WT1 mRNA show their levels associating with the degree of podocytopathy [82,83] Further investigation is needed to determine the value of lipid metabolites in urinary EVs for the prognostication of CKD

CKD: chronic kidney disease; DKD: diabetic kidney disease; EV: extracellular vesicles; GSK-3β: Glycogen Synthase Kinase 3-Beta; IgA: immunoglobulin A; mRNA: messenger ribonucleic acid; PCR: polymerase chain reaction; WT-1: Wilms Tumor-1.

## Data Availability

Not Applicable.
